# *In silico* discovery of group II intron RNA splicing inhibitors

**DOI:** 10.1021/acschembio.3c00160

**Published:** 2023-08-21

**Authors:** Olga Fedorova, Grace Arhin, Anna Marie Pyle, Aaron T. Frank

**Affiliations:** 1 Howard Hughes Medical Institute.; 2 Department of Molecular, Cellular and Developmental Biology, Yale University, New Haven, CT 06520.; 3 Biophysics Program, University of Michigan, Ann Arbor, MI 48109; 4 Department of Chemistry, Yale University, New Haven, CT 06520.; 5 Current address: Arrakis Therapeutics, Waltham, MA 02451

## Abstract

Here we describe the discovery of compounds that inhibit self-splicing in group II introns. Using docking calculations, we targeted the catalytic active site within the *O. iheyensis* group II intron and virtually screened a library of lead-like compounds. From this initial virtual screen, we identified three unique scaffolds that inhibit splicing *in vitro*. Additional tests revealed that an analog of the lead scaffold inhibits splicing in an intron-dependent manner. Furthermore, this analog exhibited activity against the group II intron from a different class: the yeast ai5γ IIB intron. The splicing inhibitors we identified could serve as chemical tools for developing group II intron-targeted antifungals, and, more broadly, our results highlight the potential of *in silico* techniques for identifying bioactive hits against structured and functionally complex RNAs.

## INTRODUCTION

Highly structured RNA molecules play pivotal roles in many important biological processes that include the regulation of transcription and translation of neighboring genes, serving as scaffolds for proteins and other RNAs, the regulation of chromatin topology, and the encoding of functional micropeptides (1). In humans, mutations and dysregulation of noncoding RNAs (ncRNAs) and coding RNAs underpin diseases such as cancer (2), Alzheimer’s disease (3), spinocerebellar ataxia (4), and fragile X syndrome (5,6). In pathogens, highly structured RNA motifs regulate biological processes that, if disrupted, can short-circuit the lifecycle of the organism. In many instances, the biological function of these functional RNA motifs is linked to their ability to sample and transit between distinct conformational states. Highly structured RNAs are particularly intriguing because they can harbor cavities that accommodate small molecule ligands, just like proteins. These ligands have the potential to modify the conformational dynamics of folded RNA elements and thus modulate their biological function. For these reasons, folded RNA structures have emerged as attractive drug targets.

High-throughput screening (HTS) can identify molecules that bind to and modulate RNA. However, screening large libraries against RNA motifs is both costly and time-consuming. As an alternative, virtual screening can rapidly identify compounds within a diverse library that are likely to bind a given RNA substructure. The best-scoring compounds can be tested via *in vitro* or *in vivo* assays. In this way, bioactive compounds can be identified without purchasing and assaying each compound in a screening library.

Although structure-based virtual screening (SBVS) has been successfully applied to protein targets for decades, its application to RNA targets has been limited. This is partly due to the lack of high-resolution 3D structures of functional RNA molecules and the challenges associated with modeling the dynamics of RNA. Despite these challenges, SBVS has been successfully applied to identify bioactive ligands that modulate the 27-nucleotide (nt) HIV-1 TAR RNA (7–9) and pseudoknot RNAs implicated in −1 frameshifting (10,11). In addition, SBVS has been applied to target the well-characterized aptamer of the purine-sensing riboswitch (12) and long non-coding RNA MALAT1(13). To date, however, SBVS has been applied to relatively small RNAs (<100-nt).

In this study, we tested whether SBVS could identify compounds that inhibit RNA splicing by targeting a self-splicing group IIC intron from the eubacterium *Oceanobacillus iheyensis* (*O.i)*. The *O.i.* intron is a 390-nt RNA for which many high-resolution crystal structures have been solved (14–16), making it a promising target for SBVS. The analogous yeast ai5γ group IIB intron has been previously established as an attractive target for discovering antifungals (17). Unlike animals, fungal organisms contain group II introns in many critical genes, making this class of ncRNA an attractive drug target. However, the lack of high-resolution structures of fungal introns precluded their use in virtual screening. Since the active site of group II introns is highly conserved, we reasoned that, by targeting the active site of the *O.i.* intron, we could discover broad-spectrum group II intron splicing inhibitors. The 390-nt *O.i.* intron represents one of the largest RNA molecules subjected to SBVS. After screening a library of 169353 compounds, we discovered a novel class of small molecules that inhibits splicing, with K_i_ values ranging from 1.5 to 30 μM in an intron-dependent manner. In this way, our study serves as a proof-of-concept for applying SBVS to target the structures of large RNA tertiary structures, highlighting its potential for the identification of small molecules that target pharmacologically relevant RNA molecules.

## RESULTS AND DISCUSSION.

### Virtual screening for the inhibitors of the *O.i.* group II intron.

**Figure 1.**
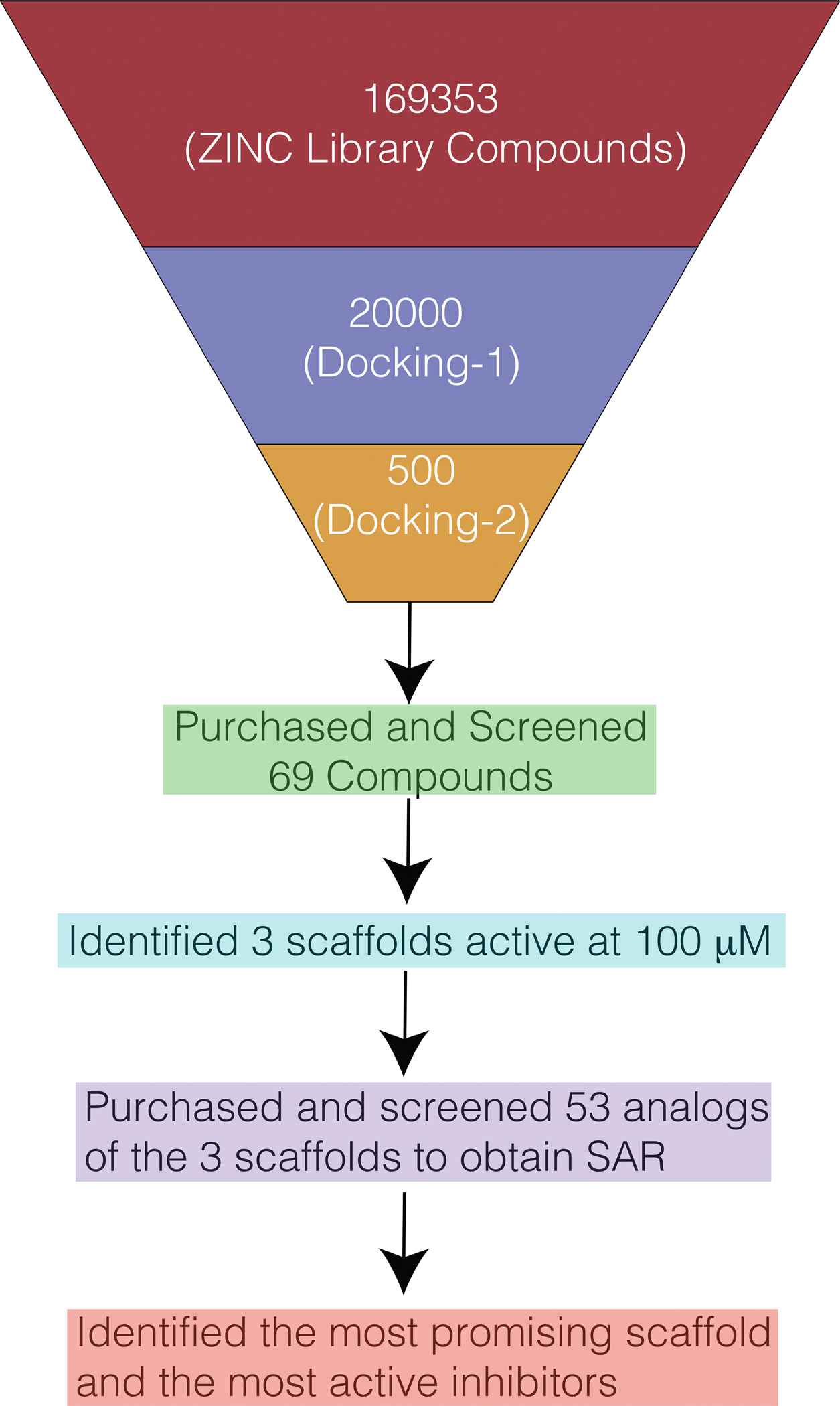
A schematic of the pipeline for the virtual screening and subsequent SAR.

**Figure 2.**
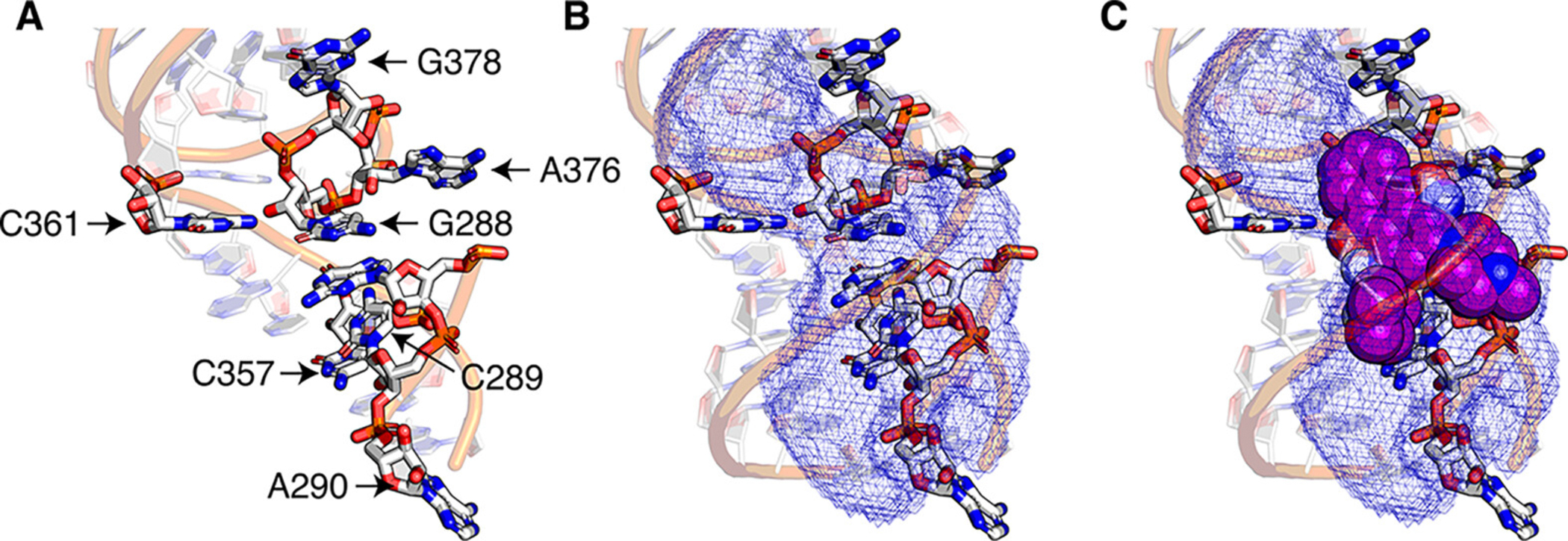
Three-dimensional architecture of the active site of the *O. iheyensis* group II intron. B, A cavity in the active site of the *O. pheresis* group II intron (left) and an original hit 1 docked into the intron active site (right).

First, we asked whether it might be possible to identify splicing inhibitors by targeting the pre-catalytic state of the *O.i.* group II intron RNA. To initiate this process, we used computational docking to carry out a virtual screen of 169353 compounds on this RNA (PDB ID: 4FAR) by targeting the catalytic site within the intron (Figures 1 and 2, Supplementary Figure 1, Methods). The catalytic site of the intron includes the catalytic triplex formed by the catalytic triad within the catalytic Domain 5 (nucleotides C358, G359 and C360, which are base-paired with G385, U384 and G383, respectively), two nucleotides of J2/3 (G288 and C289) and C377 from the two-nucleotide bulge in D5 (14) (Supplementary Figure 1). Despite the general lack of primary sequence conservation in group II introns, the active site is highly conserved, especially nucleotides G359, C360, G288 and C377, which are invariant in all group II introns. Therefore, we expected that small molecules targeted to the active site of the *O.i.* intron would potentially inhibit splicing of other group II introns as well.

**Figure 3.**
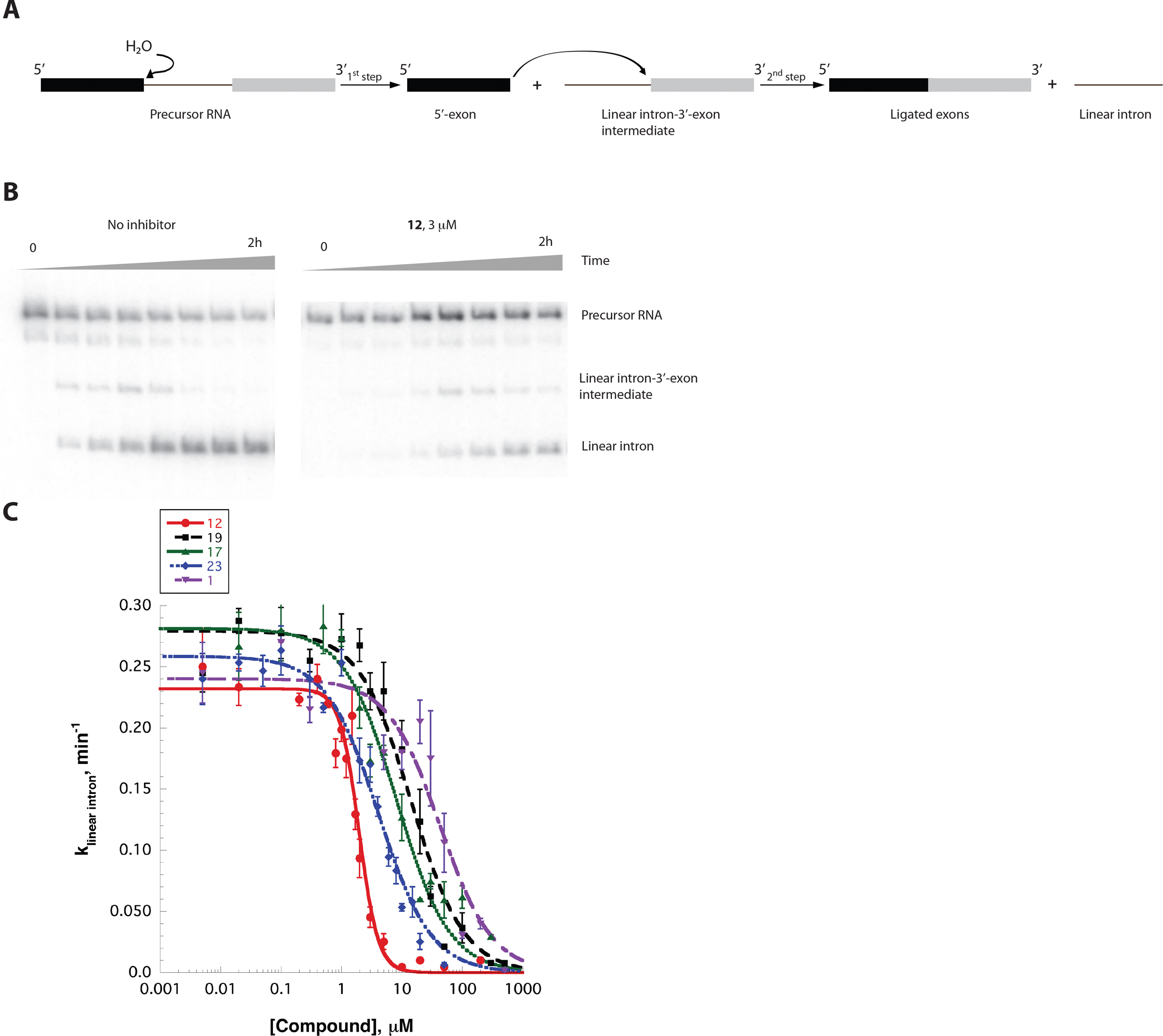
A, A self-splicing scheme for the *O iheyensis* group IIC intron. B, A PAGE analysis of representative time-courses of the *O. iheyensis* intron self-splicing in the absence (left) and in the presence (right) of 12 used to determine the splicing rate constants ($k_{obs}$). C, representative K_i_ values (Fig. 4, Supplementary Table 1) were determined by plotting the rate constants ($k_{obs}$ values) vs. inhibitor concentration. Data represent average of n=3 independent experiments. Error bars are s.e.m.

The top 500 commercially available virtual screening hits were identified, and of these, we initially purchased a diverse set of 69 compounds with the lowest (most energetically favorable) docking scores (Figure 1). We have excluded compounds that were too structurally similar or contained reactive substituents in their structure. To determine which of these hits were functional inhibitors, we tested them using a single-dose splicing inhibition assay, which monitors the rate constant for the first and second splicing steps at 100 μM compound concentration (Figure 1, Figure 3A).

Among the 69 virtual screening hits initially tested, we discovered three unique scaffolds that inhibited the first step of splicing by at least 4-fold under the conditions tested (Compounds 1, 2, and 3, Supplementary Table 1). Using a concentration-dependent splicing inhibition assay, we subsequently found that compounds 1, 2, and 3 inhibited the first step of splicing with inhibitor constant (K_i_) 6.6±1.5, 27±7, and 36±9 μM and the second step of splicing with K_i_ of 33±8, 97±20, and 39±10 μM, respectively (Supplementary Table 1).

### *In vitro* structure-activity relationship (SAR) for *O.i.* group II intron splicing inhibition.

**Figure 4.**
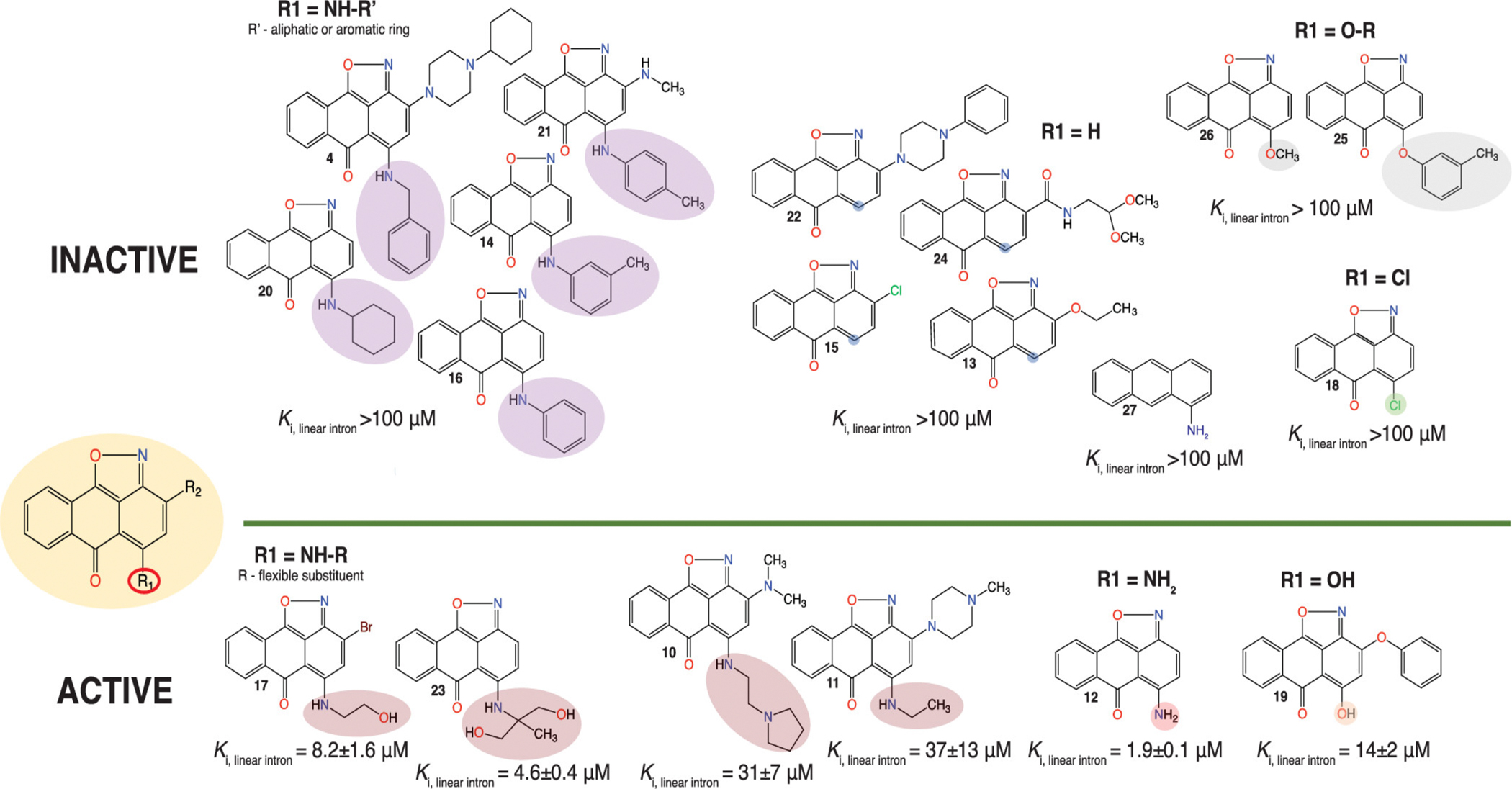
Summary of the SAR studies of the scaffold 1. In vitro splicing inhibition rate constants for the linear intron formation are shown next to the compounds. Color-coding is used to highlight changes in the functional element R1 of the scaffold as specified above each group of compounds.

Having identified three scaffolds that inhibited splicing of the target intron, we next conducted structure-activity relationship (SAR) experiments to determine the minimal pharmacophore for each scaffold, assessing the importance of various substituents, and seeking to identify more potent inhibitors. For this purpose, we analyzed 26 commercially available analogs of compound 1, 19 analogs of compound 2, and 8 analogs of compound 3. For the latter scaffold, no additional analogs were commercially available. The self-splicing assay was carried out as previously described (16) (see Materials and Methods). We determined that all available analogs for scaffold 3 and all but one analog of scaffold 2 were inactive, despite significant structural similarities to the original hits. By contrast, analogs of scaffold 1 exhibited clear SAR (Figures 3 and 4, Supplementary Table 1), causing us to focus all subsequent studies on this scaffold.

Scaffold 1 has two substituents within its third aromatic ring (Supplementary Table 1), so we first aimed to assess the importance of these groups for splicing inhibition. When we initially tested the inhibitory activity of scaffold 1 analogs, we found that the exact chemical identity of substituents within the third ring was not important for the inhibition of splicing (compare data for compounds 10 and 11, Figure 4, Supplementary Table 1). Further testing indicated that the removal of both substituents did not impede inhibitory activity, but rather significantly improved it (see data for 12, Figures 3 (B and C) and 4, Supplementary Table 1), suggesting that more active compounds would have higher ligand efficiency than the original hit (Supplementary table 1; here ligand efficiency (LE) is defined as the negative docking score normalized by the number of heavy atoms). Indeed, several compounds that are more active than the original hit also have higher ligand efficiency (compare the activity of compound 1 (LE = 1.024) to that of compounds 12 (LE = 1.725), 17 (LE = 1.519), 19 (LE = 1.462), and 23 (LE = 1.463), Figures 3 and 4. Supplementary Table 1). However, other compounds with high theoretical ligand efficiency are inactive (see data for compounds 13 (LE = 1.618), 14 (LE = 1.566), 15 (LE = 1.562), 16 (LE = 1.508), 18 (1.502), 25 (1.431), and 26 (LE = 1.345), Figure 4, Supplementary Table 1). Further analysis of the data revealed that all compounds in which the secondary amino group within the third aromatic ring of the scaffold was attached to a bulky aliphatic or aromatic ring, or replaced with H or Cl, were inactive (see data for compounds 4, 5, 7–9, 14, 16, 20, 21 and 13, 15, 18, 22 and 24, Figure 4, Supplementary Table 1). These data suggest that the amino group at the third aromatic ring of the scaffold directly interacts with the group II intron active site, possibly via hydrogen bonding.

To test the hypothesis further, we tested the activity of compound 19, in which the amino group is replaced with a hydroxyl group, which is also capable of hydrogen bonding. This compound actively inhibits splicing of the *O.i.* intron, suggesting that the hydroxyl group at this position forms productive interactions within the active site (Figures 3 and 4, Supplementary table 1). Replacement of the hydroxyl group with an 2’-O-methyl group completely abolished inhibitory activity (see data for compound 26, Figure 4, Supplementary Table 1), suggesting that the hydroxyl or amino group serves as a hydrogen bond donor, forming an interaction within the intron active site.

**Figure 5.**
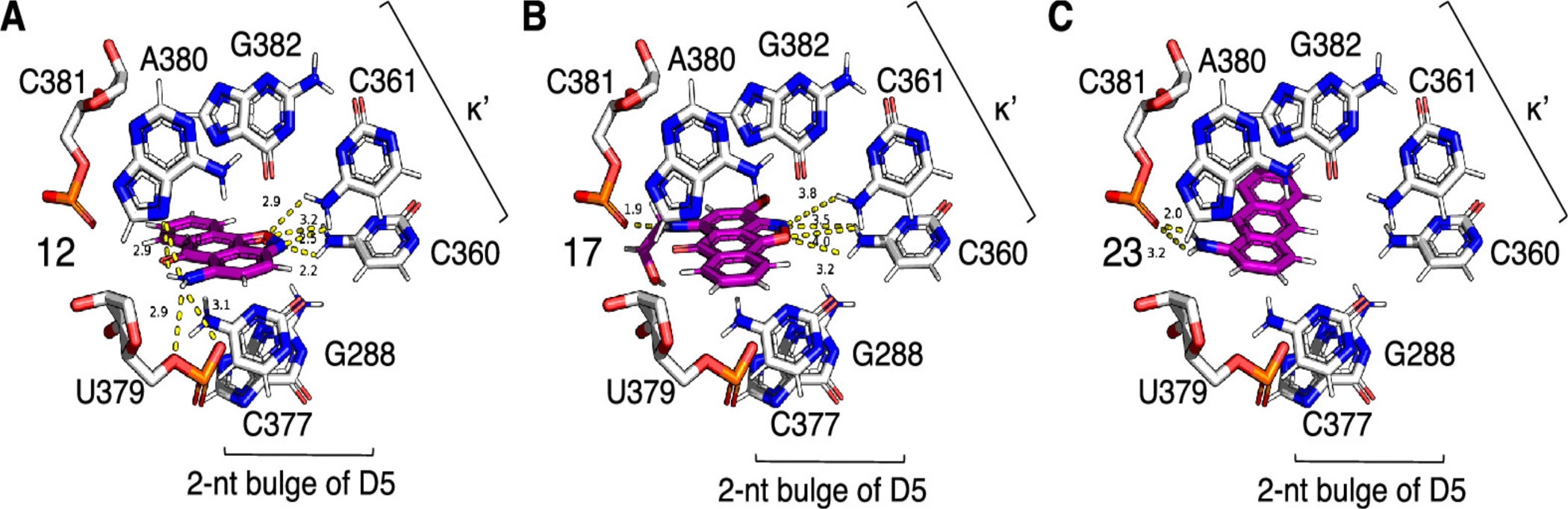
Docking into the active site of the *O. iheyensis* group II intron (A, B) of the active inhibitors and (C) inactive, aminoanthracene.

Interestingly, docking calculations predict binding modes in which the amino group of the inhibitor for polar contacts with phosphate oxygens of U379 (compound 12; Figure 5A) and A380 (compound 17; Figure 5B). The amino group of compound 12 is also predicted to form polar contacts with N7 of A380 (Figure 5A). At the same time, the oxime moiety of both inhibitors appears to be engaged in polar contacts with the exocyclic amino groups of C360 and C361 (Figure 5A, B). However, the orientation of pose 17 in the active site is flipped relative to that of 12. It appears that the bonding of the oxime moiety contributes to activity as much as the primary or secondary amino group. Removal of this moiety, resulting in a simple aminoanthracene (compound 27), completely abolishes inhibitory activity (Supplementary table 1); the predicted binding mode suggests that the loss in inhibitory activity might be due to the loss of polar contacts with the exocyclic amino groups of C360 and C361 (Figure 5C).

### Active group IIC intron inhibitor affects splicing of the ai5γ group IIB intron *in vitro*

**Figure 6.**
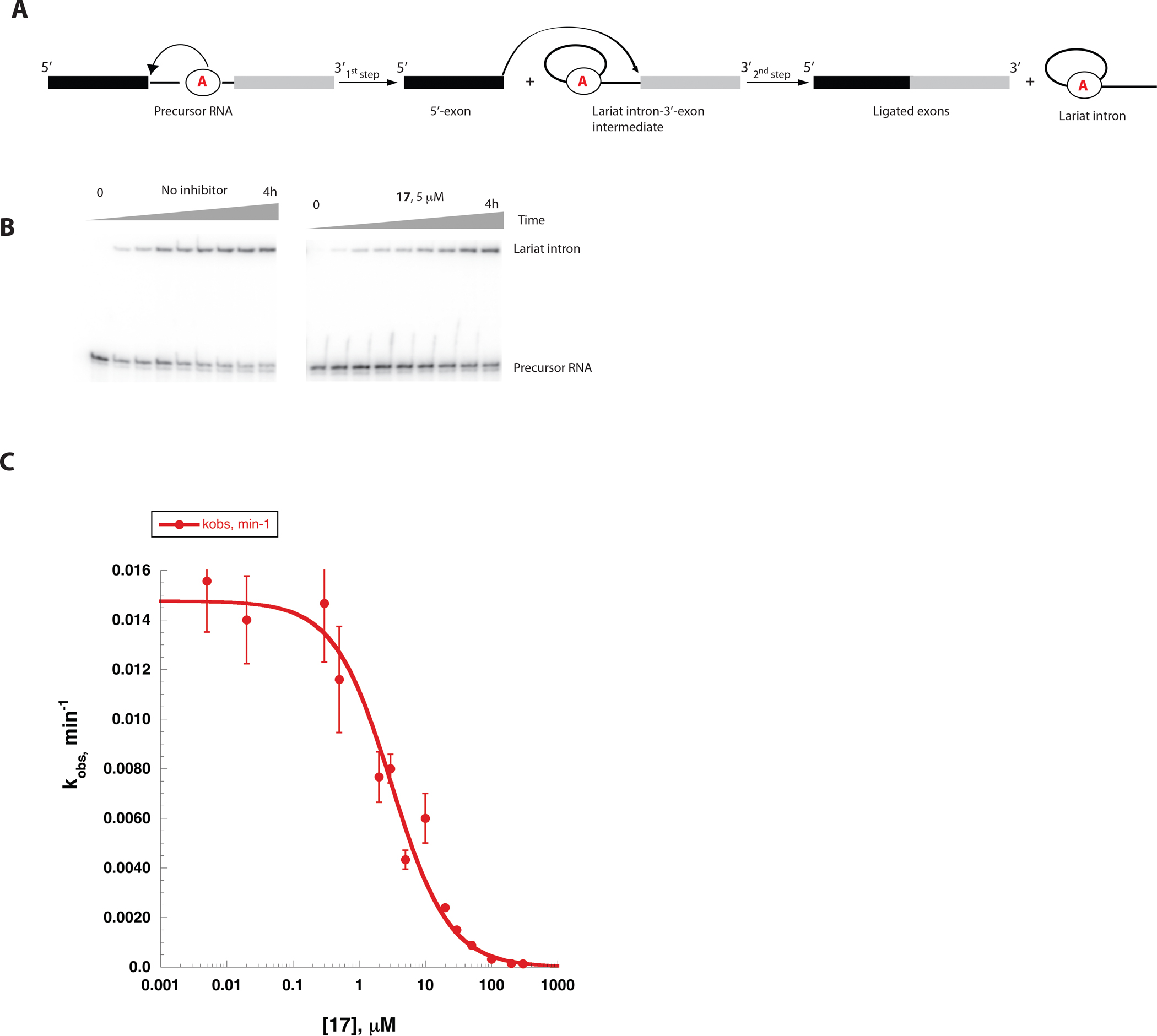
A, A self-splicing scheme for the ai5γ group IIB intron. The branch-point adenosine is highlighted in a black circle. B, A PAGE analysis of representative time-courses of the ai5γ intron self-splicing in the absence (left) and in the presence (right) of the inhibitor 17 used to determine the splicing rate constants ($k_{obs}$). C, The K_i_ value for 17 was determined by plotting the rate constants ($k_{obs}$ values) vs. inhibitor concentration. Data represent average of n=3 independent experiments. Error bars are s.e.m.

Although there are substantial differences in the overall architecture of group II intron subclasses, the tertiary structure of their cores are phylogenetically conserved (18), and their active sites are quite similar (Supplementary figure 3)(14,19). Given these parallels, we asked whether group IIC intron splicing inhibitors can also inhibit splicing of a IIB intron. Inhibitors that can function across group II intron families would be of interest given that self-splicing introns are required for metabolism of pathogenic fungi, for which new antimicrobials represent a major unmet medical need (20). To evaluate pan-splicing capability of the compounds, we tested several inhibitors of the *O.i.* intron in a self-splicing reaction of the *Saccharomyces cerevisiae* (*S.c.*) Group IIB ai5γ intron, which is a genetically and biochemically well-characterized system (21–23). Unlike the *O.i.* intron, the ai5γ intron splices via a different mechanism that involves branching during the first step, resulting in the excision of a lariat intron (Figure 6A). We found that one of the tested inhibitors, compound 17, actively inhibited splicing of the ai5γ group IIB intron with the K_i_ value of 3.1±0.6 μM (Figure 6 B, C). Remarkably, this K_i_ value is close to that observed for the first step of splicing of the *O.i.* group IIC intron (3.6±0.6 μM). This suggests that targeting the conserved active site of a specific group intron can aid in discovering compounds that are broadly inhibitory of all group II intron subtypes.

### Selective inhibition of the *O.i.* and ai5γ introns by compound 17 *in vitro*.

**Figure 7.**
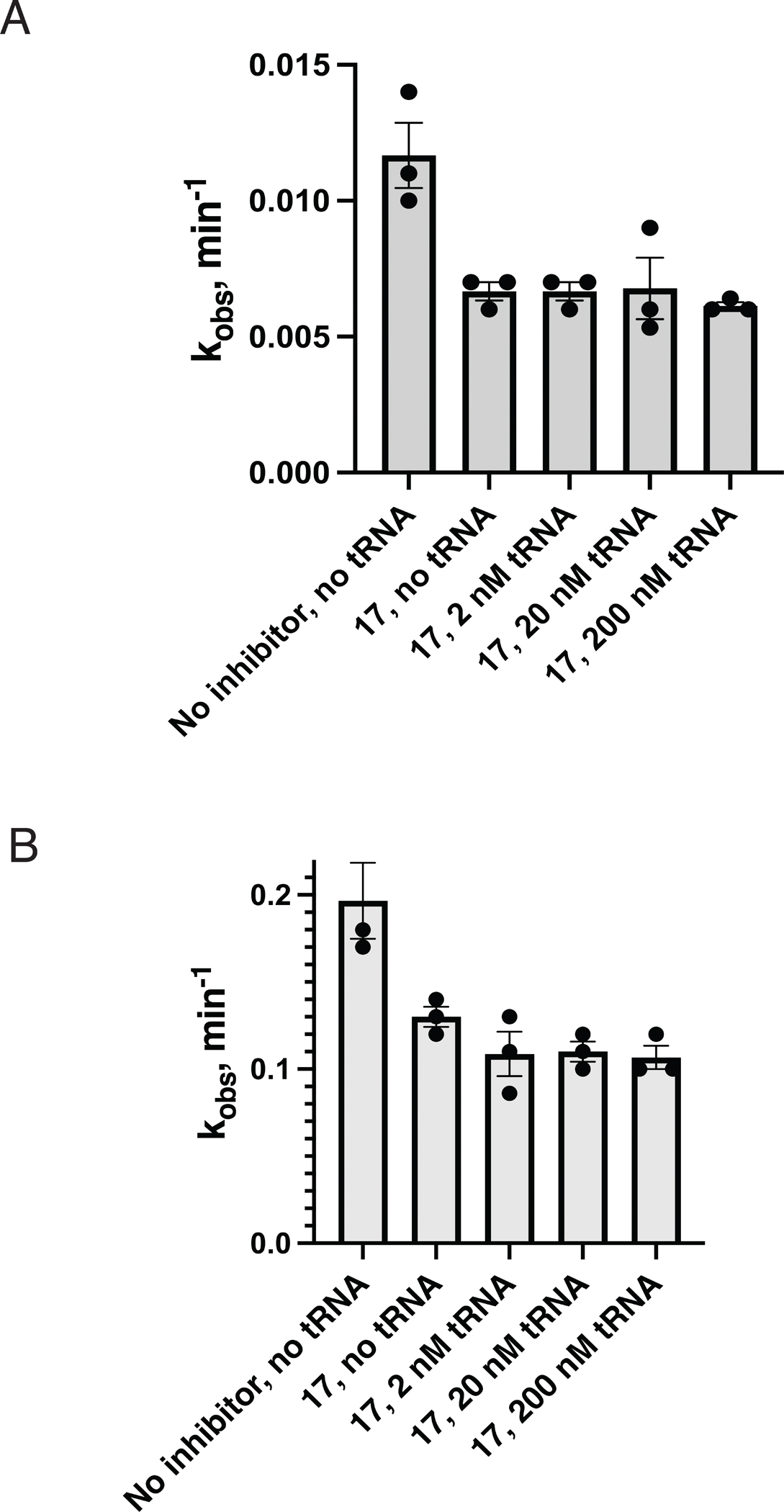
Inhibition of the *O.i.* (A) and ai5γ (B) group II intron splicing by compound 17 in the presence of various concentrations of yeast tRNA^Phe^. Data represent the average of n=3 independent experiments, error bars are s.e.m.

Since Compound 17 inhibits both *O.i*. group IIC intron and ai5γ group IIB intron, it was important to determine whether this compound selectively inhibits group II intron splicing. For this purpose, we have tested inhibition of splicing of both introns by 17 in the presence of varying concentrations of yeast tRNA^Phe^. Various tRNA molecules are frequently used as selectivity controls for RNA targeting by small molecules (24), because their tertiary architecture incudes many common RNA structural elements, for example base triples, coaxially stacked helices, U-turn motifs and kissing loops (24). Nevertheless, we found that even a 100-fold excess of the tRNA^Phe^ over the intron RNA (2 nM) did not affect inhibition of splicing of either *O.i.* or ai5γ intron by compound 17 (Fig. 7). These data suggest that compound 17 is not a non-specific RNA binder, but it selectively inhibits splicing of group II introns from two different classes.

### Selective inhibition of fungal growth

To test whether compound 17 selectively inhibits yeast growth under conditions that require intron splicing, we examined *S.c.* growth in the presence of various concentrations of compound 17 in dextrose (YPD) and glycerol (YPG) media (Table 1). Because dextrose in YPD is a fermentable carbon source for *S.c*., respiration is not required for yeast growth in this medium and splicing inhibition is therefore not expected to affect growth. By contrast, glycerol in the YPG medium is a non-fermentable carbon source and *S.c.* growth in this medium requires respiration (21). Inhibition of ai5γ intron splicing in YPG medium should therefore be detrimental to yeast growth. Using inactive compound 15 as a control, we found that neither compound 15 nor compound 17 affect yeast growth in fermentable YPD medium (Table 1). Strikingly, compound 17 inhibited the growth of *S.c.* in the nonfermentable YPG medium, while inactive compound 15 had no effect. These data indicate that compound 17 adversely affects yeast respiration, possibly by inhibiting splicing of the ai5γ intron.

To determine whether *S.c* growth inhibition by compound 17 is selectively caused by its inhibition of the ai5γ intron and not some other intron or cellular target, we studied the effect of compound 17 on the growth of a *S.c.* strain in which all introns within the Cox1 gene have been removed (Cox1 intronless strain) (25). Consistent with our model for inhibition, we found that compound 17 does not affect the growth of the Cox1 intronless strain in either YPD and YPG media (Table 1). Collectively, these data indicate that compound 17 selectively inhibits respiration and growth of WT *S.c.* by blocking ai5γ group II intron splicing. This is consistent with our results suggesting that compound 17 selectively inhibits splicing of both *O.i.* and ai5γ introns in the presence of excess tRNA.

## CONCLUSION

In this study, we demonstrated that de novo virtual screening could be used to identify potent, bioactive small molecules that modulate the function of a folded RNA with tertiary structure (in this case, a 390-nt group IIC intron), ultimately resulting in bioactive inhibitors. The virtual screen predicted several compounds that displayed inhibition constants in the low micromolar range, comparable with the activity of compounds previously identified by direct small molecule HTS (17). Moreover, by specifically targeting the conserved active site of the intron, we identified a compound that inhibits group II introns from multiple subclasses, resulting in potent, selective splicing inhibitors of group II introns in biochemical assays and in living cells. The virtual screening strategy focused on targeting the intron’s active site, where inhibitors may bind and disrupt conformational dynamics or interactions that are critical to the self-splicing reaction. It is important to note that these molecules have some pharmacological liabilities despite their apparent potency. For example, the fused ring system resembles mitoxantrone, a common intercalator and topoisomerase inhibitor. The quinone moiety might result in off-pathway redox effects. Nevertheless, it should be emphasized that these compounds do not inhibit cell growth in an intronless system (where splicing of this exact intron is not required), suggesting that they lack off-pathway effects and they are target specific.

Going forward, as high-resolution structures provide detailed information on the exact binding site of the compounds, it will ultimately be possible to formulate a more precise mechanism of action. However, the discovery and bioactivity of these inhibitors highlights the fact that virtual screening is a promising alternative to experimental HTS screening for early-stage hit identification against functional RNA tertiary structures.

## Materials and Methods

### Virtual screen

Virtual screening was carried out using a curated library of 169353 docking-ready compounds from the ZINC database (26,27). The randomly selected compounds represent a diverse collection of drug-like molecules with hydrogen bond donors (HBD)<5, hydrogen bond acceptors (HBA) 1<10, molecular weight (MW)<500, and octanol-water partition coefficient (logP)<5. The RNA coordinates used for rigid docking were extracted from the crystal structure of *Oceanobacillus iheyensis (O.i.*) group IIC intron (PDB ID: 4FAR). Small molecule docking was focused on a pocket near the group-II intron active site (Figure 2). A 2-phase docking protocol was carried out using rDock software (28). In phase 1, the entire library was docked against 4FAR using the fast rDock score term, which does not account for solvation effects. In phase 2, the 20000 compounds with the lowest docking score were redocked using the slower rDock score term that does account for solvation effects. Whereas in phase 1, 50 poses were generated for each compound and the pose with the lowest docking score was identified, in phase 2, 1000 poses were generated for each compound, and then the pose with the lowest docking score was identified. The 500 compounds from phase 2 with the lowest docking were identified. From this small collection, 122 commercially available compounds were purchased and tested using group II intron self-splicing assay (see below). The pose of the active compounds was predicted by filtering the rDock poses using the tool RNAPosers (29), which is a collection of machine learning classifiers that have been previously shown to enhance pose prediction accuracy.

### Yeast strains

*S. cerevisiae* (*S.c.)* strains (Wild-type: NP40–36a, mtDNA intronless: XPM460) were a gift from Dr. Thomas Fox (Dept. of Molecular Biology and Genetics, Cornell University).

### RNA preparation.

Internally ^32^P-labeled *O.i.* and ai5γ (SE) precursor RNAs were *in vitro* transcribed in the presence of ^32^P-α-UTP from plasmids pOiA (14) and pAS10 (30) that were linearized with ClaI or HindIII restriction enzymes, respectively, as previously described (31). Transcription was carried out for 90 minutes and RNAs were purified on a 5% denaturing polyacrylamide gel as described (31,32).

### Determination of the small molecule inhibition constants for group IIC and group IIB intron splicing

Internally labeled SE or *O.i.* RNA (2 nM) was incubated with various concentrations of small molecule inhibitor under near-physiological conditions (in 50 mM MOPS, pH 7.5, 8 mM MgCl_2_, 150 mM (NH_4_)_2_SO_4_, 30^o^C for the SE RNA; and in 50 mM MOPS, pH 7.5, 5 mM MgCl_2_, 150 mM KCl, 37 ^o^C for the *O.i* RNA). Aliquots of the reaction mixture were withdrawn at different time points, quenched in a formamide buffer (82 % (v/v) deionized formamide, 0.16 % (w/v) xylene cyanol (XC), 0.16 % (w/v) bromophenol blue (BB), 10 mM EDTA, pH 8.0) and analyzed on a 5% denaturing polyacrylamide gel as previously described (15). For the SE RNA, the fraction of the lariat intron was plotted versus time and fit to a single-exponential equation to determine the first-order rate constants ($k_{obs}$). For the *O.i.* intron, $k_{obs}$ values were determined for all reaction products using Global Explorer software (KinTek). The latter were plotted against the concentration of inhibitor and fit to the equation for non-competitive inhibition to determine $K_{i}$ values:

$$k_{obs}=\frac{k_{max}}{\left(1+\frac{\left[I\right]}{K_{i}}\right)},$$

where $k_{obs}$ and $k_{max}$ are the first order rate constants measured in the presence and in the absence of the inhibitor, respectively, [I] is the concentration of the inhibitor and $K_{i}$ is the inhibition constant. Experiments were performed in triplicate, data represent average ± s.e.m.

### Splicing inhibition in the presence of excess tRNA

Inhibition of the *O.i.* and SE RNA splicing by compound 17 in the presence of excess tRNA was monitored essentially as previously described (17). Briefly, the internally labeled *O.i.* or SE (ai5γ) RNA (2 nM) and unlabeled tRNA^Phe^ at different concentrations (2, 20 or 200 nM) were separately preincubated in 50 mM MOPS, pH 7.5, 100 mM monovalent salt (KCl for the *O.i*. intron reaction mixtures or (NH_4_)_2_SO_4_ for the ai5γ intron reaction mixtures) and MgCl_2_ (2 mM for the *O.i.* intron, 5 mM for the ai5γ intron and for the tRNA) at 37 ^o^C (*O.i.* intron reaction mixtures) or 42 °C (ai5γ intron reaction mixtures) for 20 min. The intron and tRNA mixtures were combined, 17 was added to the final concentration of 3 μM for the ai5γ reaction mixtures and 5 μM for the *O.i.* reaction mixtures. Reaction were initiated by adding salts to the final concentrations of 150 mM KCl and 5 mM MgCl_2_ for the *O.i.* reaction mixtures and 500 mM (NH_4_)_2_SO_4_ and 100 mM MgCl_2_ for the ai5γ reaction mixtures. Aliquots were withdrawn at different time points, quenched and analyzed on 5% denaturing polyacrylamide gels as described above.

### Small molecule inhibition of *S.c.* growth in YPD and YPG media

Inhibition of yeast growth in the presence of small molecules was assayed either in liquid YPD media (BD Bacto Yeast Extract, BD Bacto Peptone, 2% glucose) or in YPG media (BD Bacto Yeast Extract, BD Bacto Peptone, 3% glycerol), essentially as described for the antifungal MIC assays using the guidelines from the Clinical and Laboratory Standards Institute (17). At the beginning of each experiment, a fresh stock solution in DMSO was prepared for each compound (3.2 mg/ml for Amphotericin B and 6.4 mg/ml for each test compound). This solution (100 μl) was pipetted into the first well of a 12-well row on a 96-well plate (compound preparation plate). The subsequent eleven wells in this row were filled with 50 μl of DMSO for serial dilution. Concentrated compound solution (50 μl) was withdrawn from the first well, transferred to the second well and mixed. This process was repeated for eight subsequent wells, resulting in a 1:2 serial dilution of the stock into DMSO for wells 1–10. Wells 11 and 12 were used as the no-compound control and sterility control, with no inoculum added, respectively, therefore they contained only DMSO. Next, 2.5 μl from each well of the compound preparation plate was transferred to the wells of a separate 96-well plate (media dilution plate), and mixed with 122.5 μl of YPD or YPG medium, resulting in a set of serial dilutions of compound into growth media in a volume of 125 μl. Then 50 μl from each of the wells of the media dilution plate were transferred onto a third 96-well plate (the assay plate) and mixed with an equal volume (50 μl) of freshly prepared yeast inoculum in YPD or YPG medium resulting in the final inoculum concentration of 0.5×10^3^ to 2.5×10^3^ colony forming units per 1 mL (wells 1–11), or YPD/YPG alone (sterility control, well 12) and DMSO concentration in each well was 1%. The final compound concentration range of the assay was 0.125 – 64 μg/ml for test compounds and 0.0625 – 32 μg/ml for Amphotericin B. Plates were incubated at 30^o^C and visually analyzed for turbidity after 24h of incubation for the YPD medium and 2 weeks for the YPG medium. The lowest compound concentration at which no growth was apparent is reported as the MIC (Table 1). All experiments were performed in triplicate.

## Supplementary Material

SI

## Figures and Tables

**Table 1.** Small molecule inhibition of growth of the wild type and intronless strains of *S. cerevisiae* in YPD and YPG media. The results represent average of four independent experiments, errors are s.e.m.

Compound	MIC, μg/ml			
	YPD medium		YPG medium	
	WT strain	Intronless strain	WT strain	Intronless strain
AF 112	>64	>64	12±2	>64
AF 109	>64	>64	>64	>64
Amphotericin B	0.5	0.5	0.5±0.14	1.0±0.3
