# Supplementary material for: In silico discovery of group II intron RNA splicing inhibitors
Source: ACS Chem Biol. Author manuscript; Available in PMC 2026 Mar 31. (PMC13036695; doi:10.1021/acschembio.3c00160)
Supplement: SI [file NIHMS2077241-supplement-SI.pdf]

In silico discovery of group II intron RNA splicing inhibitors

Supporting information

Olga Fedorova[1,2], Grace Arhin[3], Anna Marie Pyle[1,2,4*], and Aaron T. Frank[3,5*]

[1]Howard Hughes Medical Institute.

[2]Department of Molecular, Cellular and Developmental Biology, Yale University, New Haven, CT 06520.

[3]Biophysics Program, University of Michigan, Ann Arbor, 48109

[4]Department of Chemistry, Yale University, New Haven, CT 06520.

[5]Current address: Arrakis Therapeutics, Waltham, MA, 02451

*Correspondence to: anna.pyle@yale.edu and afrank@arrakistx.com

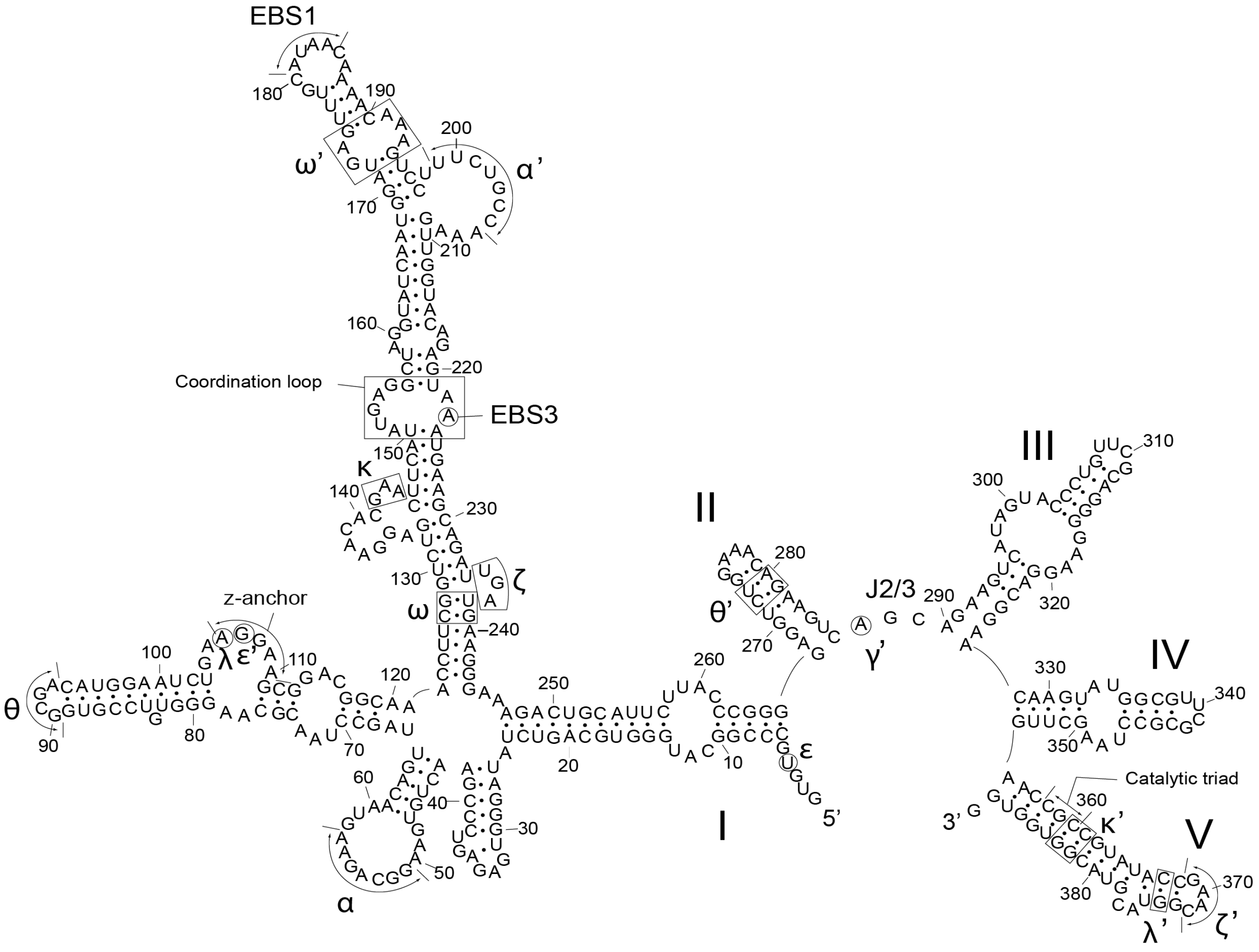

Figure S1. The secondary structure of the *O. iheyensis* group IIC intron.

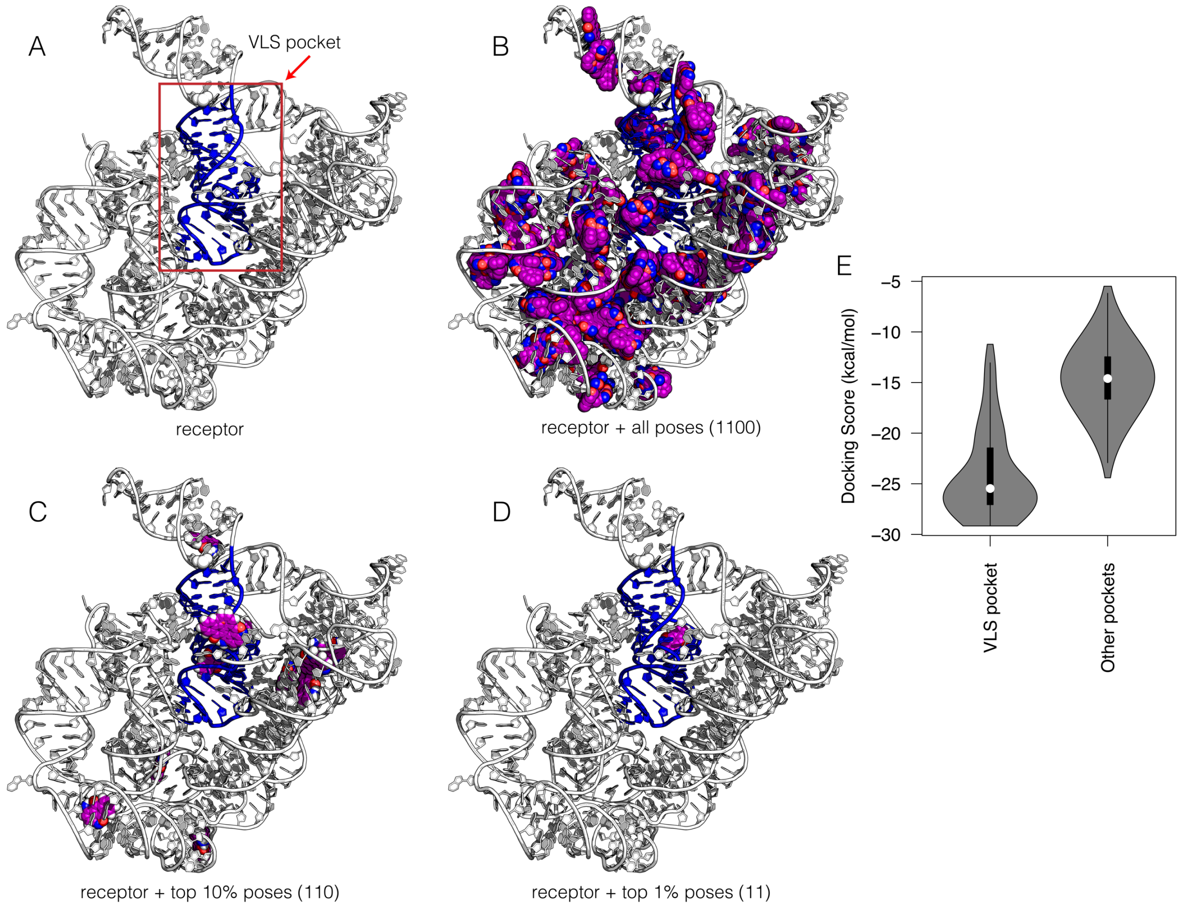

Figure S2. To determine if the inhibitors found by carrying out a virtual ligand screening (VLS) against a pocket near the active site of the group-II intron can bind to other sites, we identified the top 10 pockets on the entire surface and redocked compound 17; we generated 1000 poses in these pockets and compared them to 100 poses generated in the VLS pocket. Shown here is the receptor with the VLS pocket highlighted (A), the receptor with all the docked poses (B), the top 10% of poses (C), the top 1% of poses (D), and the distribution of docking scores for poses targeting the VLS pocket and alternative pockets on the RNA (E). According to our docking results, the VLS pocket is preferred relative to other sites on the surface of the group-II intron.

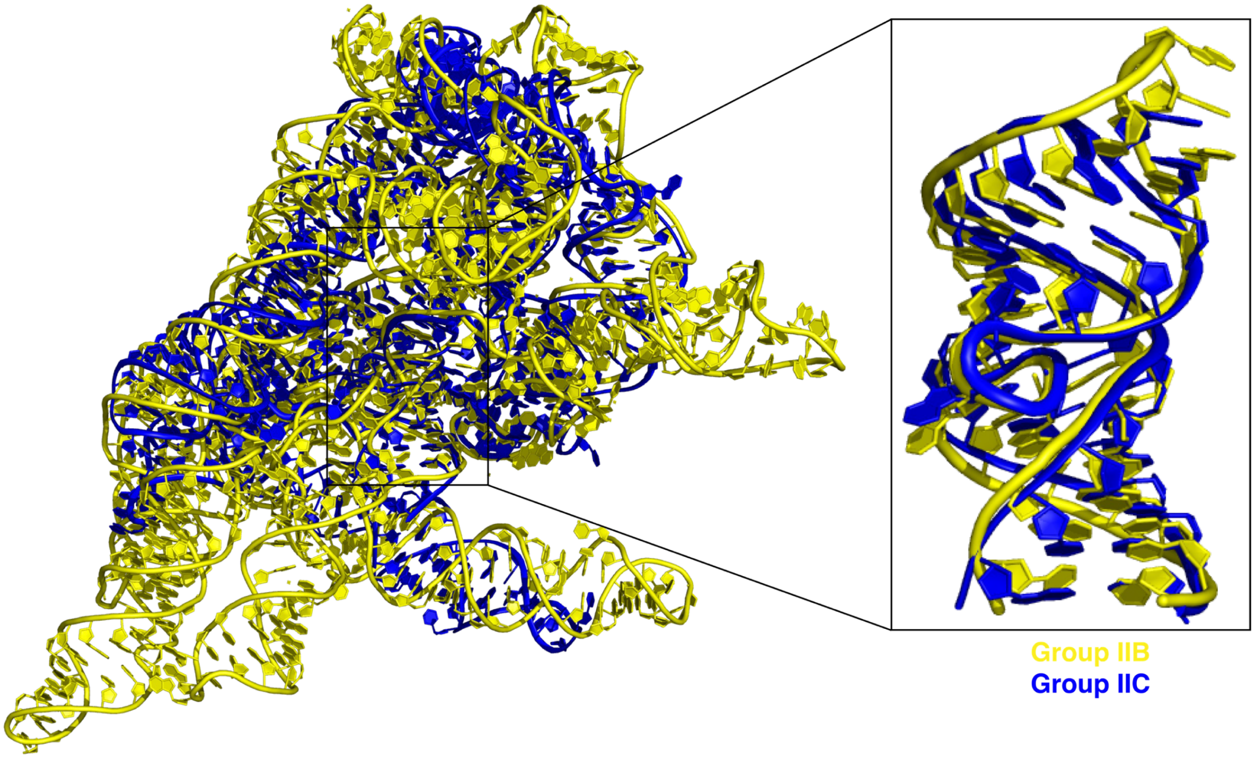

Supplementary Figure 3. Overlay comparing the structure of group IIB (PDB ID: 4R0D) and IIC (PDB ID: 4FAR) introns.

Table S1. Structure-activity relationships of representative *O. iheyensis* Group II intron inhibitors.

Compound	Structure	MW	Docking score from rDock (ΔG), kcal/mol	Ligand efficiency (LE), kcal/mol	Ki first step, μM	Ki second step, μM	Internal designation
1	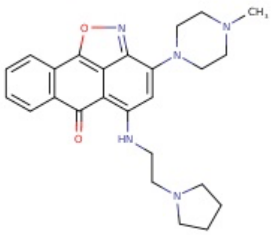	431.540	-32.778	1.024	6.6±1.5	33±8	AF 51
2	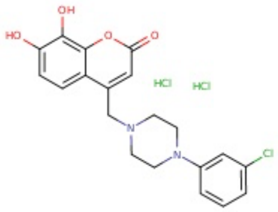	459.750	-36.464	1.351	27±7	97±20	AF 31
3	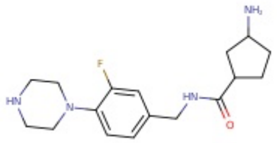	320.412	-39.340	1.710	36±9	39±10	AF 55
4	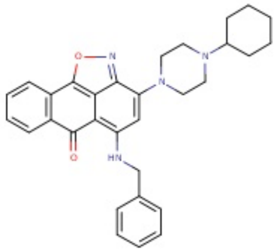	492.620	-34.425	0.930	>100[1]	>100	AF 67

5	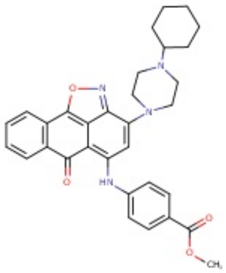	536.630	-24.322	0.608	>100	>100	AF 69
6	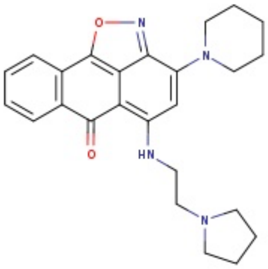	416.520	-31.736	1.024	14±3	38±6	AF 70
7	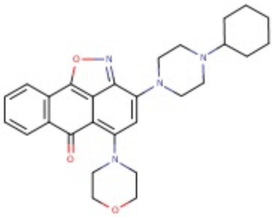	472.590	-27.452	0.784	>100	>100	AF 72
8	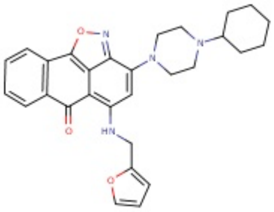	482.580	-34.149	0.949	>100	>100	AF 73

9	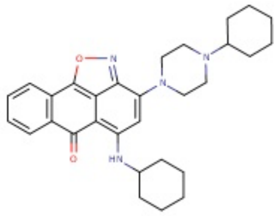	484.640	-31.957	0.888	>100	>100	AF 75
10	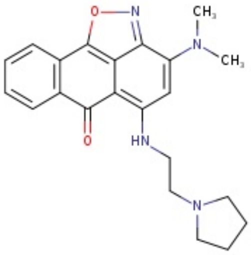	376.460	-28.022	1.001	13±2	31±7	AF 77
11	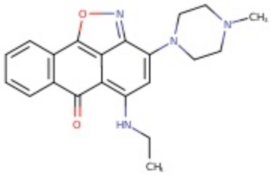	362.430	-31.312	1.160	21±2	37±13	AF 78
12	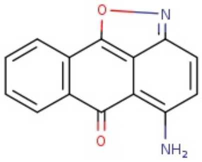	236.230	-31.052	1.725	1.53±0.03	1.9±0.1	AF 106
13	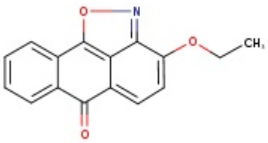	265.268	-32.365	1.618	>100	>100	AF 107

14	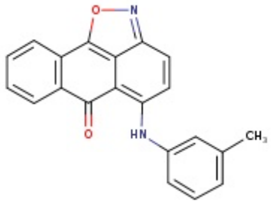	326.355	-39.152	1.566	>100	>100	AF 108
15	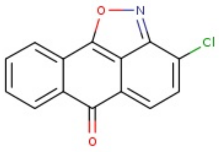	255.660	-28.113	1.562	>100	>100	AF 109
16	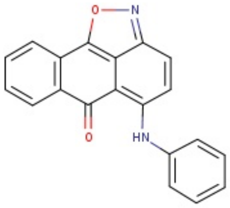	312.328	-36.185	1.508	>100	>100	AF 110
17	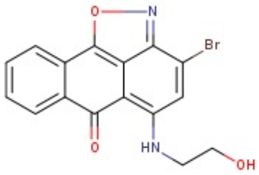	359.179	-33.421	1.519	3.6±0.6	8.2±1.6	AF 112
18	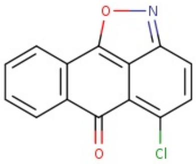	255.660	-27.043	1.502	>100	>100	AF 113
19		329.311	-36.541	1.462	12±2	14±2	AF 114

	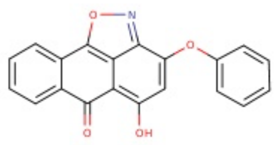						
20	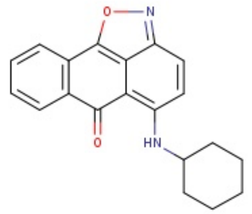	318.376	-28.028	1.168	>100	>100	AF 116
21	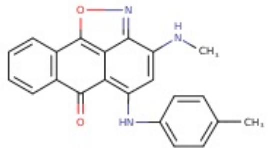	355.397	-31.248	1.157	>100	>100	AF 117
22	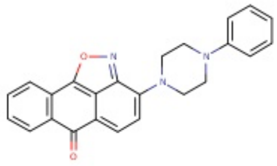	381.435	-23.752	0.819	>100	>100	AF 118
23	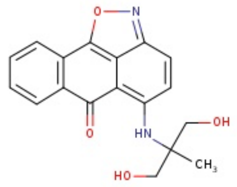	324.336	-35.123	1.463	3.8±0.2	4.6±0.4	AF 119
24	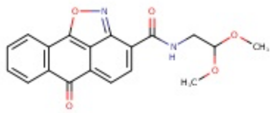	352.346	-24.839	0.955	>100	>100	AF 120

25	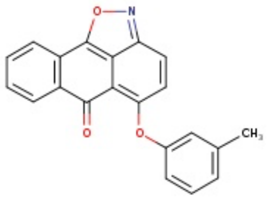	327.339	-35.775	1.431	>100	>100	AF 121
26	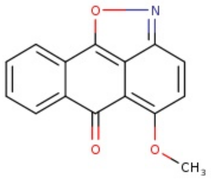	251.241	-25.549	1.345	>100	>100	AF 122
27	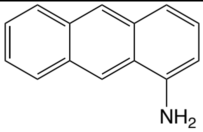	193.240	-27.777	1.852	>100	>100	AF 123

[1]No activity was detected between 5 nM and 100 μM of the compound.
